# Unveiling heterogeneity of hysteresis in perovskite thin films

**DOI:** 10.1186/s11671-024-03996-9

**Published:** 2024-03-18

**Authors:** Zhouyiao Zou, Haian Qiu, Zhibin Shao

**Affiliations:** 1 Industrial Training Center, Shenzhen Polytechnic University, Shenzhen, 518055 Guangdong China; 2 Physics Laboratory, School of Undergraduate Education, Shenzhen Polytechnic University, Shenzhen, 518055 Guangdong China

**Keywords:** Hysteresis, Perovskite thin film, Photoconductive atomic force microscopy, Charge transport, Ion migration

## Abstract

The phenomenon of current–voltage hysteresis observed in perovskite-based optoelectronic devices is a critical issue that complicates the accurate assessment of device parameters, thereby impacting performance and applicability. Despite extensive research efforts aimed at deciphering the origins of hysteresis, its underlying causes remain a subject of considerable debate. By employing nanoscale investigations to elucidate the relationship between hysteresis and morphological characteristics, this study offers a detailed exploration of photocurrent–voltage hysteresis at the nanoscale within perovskite optoelectronic devices. Through the meticulous analysis of localized *I*–*V* curve arrays, our research identifies two principal hysteresis descriptors, uncovering a predominantly inverted hysteresis pattern in 87% of the locations examined. This pattern is primarily attributed to the energetic barrier encountered at the interface between the probe and the perovskite material. Our findings underscore the pronounced heterogeneity and grain-dependent variability inherent in hysteresis behavior, evidenced by an average Hysteresis Index value of 0.24. The investigation suggests that the localized hysteresis phenomena cannot be exclusively attributed to either photocharge collection processes or organic cation migration at grain boundaries. Instead, it appears significantly influenced by localized surface trap states, which play a pivotal role in modulating electron and hole current dynamics. By identifying the key factors contributing to hysteresis, such as localized surface trap states and their influence on electron and hole current dynamics, our findings pave the way for targeted strategies to mitigate these effects. This includes the development of novel materials and device architectures designed to minimize energy barriers and enhance charge carrier mobility, thereby improving device performance and longevity. This breakthrough in understanding the microscale mechanisms of hysteresis underscores the critical importance of surface/interface defect trap passivation in mitigating hysteretic effects, offering new pathways for enhancing the performance of perovskite solar cells.

**Supplementary Information:**

The online version contains supplementary material available at 10.1186/s11671-024-03996-9.

## Introduction

The certified power conversion efficiency of perovskite solar cells has exceeded 26% [1]. Nevertheless, the pervasive occurrence of current–voltage hysteresis effects within these devices hinders further optimization and stability enhancement [2–5]. The exploration of the underlying physical mechanisms to inform device design and optimization holds significant scientific significance and practical importance, as it aims to eliminate this effect and enhance the operational stability of the devices.

In 2014, the Snaith group conducted a systematic study on the normal hysteresis phenomenon observed in perovskite solar cells, particularly those with three common device structures [3]. The Snaith group’s findings indicate that the hysteresis phenomenon is primarily influenced by the perovskite light-absorbing layer and the interface between the light-absorbing layer and the transport layer. Subsequent to the initial findings, research groups led by Huang [6], Sargent [7], and Huettner [8] conducted studies on the role of fullerene in mitigating the hysteresis effect. The research groups confirmed that there are numerous charge defect states present in perovskite films, which are primarily located at the crystal surface and grain boundaries. Additionally, some scholars have proposed that the ferroelectricity and polarization effects of perovskite materials under illumination are significant factors contributing to the hysteresis effect [9–12]. The observed ion migration in perovskite thin films is thought to be another contributing factor to the hysteresis effect [13–16]. It is believed that ion migration is associated with the formation of defects in perovskite crystals, with the primary mobile ions being halide ions or vacancies, as well as organic cations [17–24]. In addition to mechanisms mentioned above, other potential explanations include the presence of defects [25], charge trapping and de-trapping processes [26, 27], accumulation of interfacial charge [28, 29], as well as compositional variations [30, 31] in the perovskite thin films.

Despite the numerous studies conducted on the hysteresis observed in perovskite solar cell devices, the scientific community has yet to reach a consensus on the underlying mechanisms responsible for it. Few studies have been conducted to investigate the conditions leading to the occurrence of normal and anomalous hysteresis effects, as well as the physical mechanisms involved in their conversion. Besides, the majority of existing studies have primarily focused on the macroscopic characterization of photovoltaic devices, where the *I*–*V* characteristic curves provide an average representation of photovoltaic responses across the microscopic scale. However, these studies fail to capture the spatial variations in photovoltaic responses and hysteresis effects at the microscopic level.

The preparation of perovskite polycrystalline thin films typically involves a solution process using precursor materials, leading to grain sizes that span from several hundred nanometers to the micrometer scale. At the microscopic level, the surface morphology of perovskite polycrystalline thin films plays a crucial role in determining the operational mechanisms of photovoltaic devices. Previous studies have consistently demonstrated that perovskite polycrystalline grains possess distinct characteristics in terms of photovoltaic response [32, 33], photo-induced luminescence [34], crystal orientation [35], crystallinity [36], carrier lifetime [37], and defect state density [38]. These differences at the microscopic scale cannot be intuitively reflected in macroscopic device measurements. Characterization of nanoscale *I*–*V* characteristic curves can intuitively display the spatial distribution of photovoltaic responses and hysteresis effects on the film surface, facilitating the exploration of the relationship between hysteresis effects and factors such as ion migration, interface barriers, and charge accumulation through other scanning probe microscopy techniques. This further reveals the microscopic origins of the hysteresis effect. Consequently, the examination of hysteresis effects from a nanoscale perspective represents a crucial approach for comprehending the underlying mechanisms of hysteresis. So far, Due to the limitations of experimental means, related research is relatively scarce.

Using a conductive probe as the upper electrode, Photoconductive Atomic Force Microscopy (pc-AFM) technology scans and collects photocurrent generated by the sample under illumination [33, 34, 39, 40]. Additionally, it records the *I*–*V* characteristic curves of the sample surface under scanning voltage. Building upon Pc-AFM, a point-by-point *I*–*V* (PPIV) scanning technique has been recently developed to collect *I*–*V* characteristic curves at each pixel point [41–43]. Following data processing and programming in the later stage, an image of the photovoltaic parameters of the perovskite film surface is reconstructed. During PPIV scanning, after collecting the *I*–*V* curves at a pixel point, the probe is lifted to a set height and then moved to the next pixel point, effectively avoiding damage and wear to the sample surface by the probe.

By employing nanoscale mapping techniques, we reveal the heterogeneity of photocurrent voltage hysteresis across different regions of perovskite thin films via mapping of two hysteresis descriptors, highlighting the influence of interfacial energetic barriers and localized surface trap states. Our findings demonstrate that hysteresis behavior is not uniform, suggesting targeted approaches for mitigating its effects could significantly enhance device performance. This work advances our understanding of hysteresis mechanisms at the nanoscale and offers practical insights for the development of more stable and efficient perovskite solar cells.

## Methods and materials

### Perovskite thin film fabrication

Indium tin oxide (ITO)-coated glass substrates underwent a cleaning regimen, initiated with immersion in a detergent solution, followed by sequential ultrasonication treatments in deionized water, acetone, and isopropanol for a duration of 10 min each. Subsequent to the cleaning process, the substrates were dried utilizing nitrogen gas and subjected to UV-ozone treatment for 15 min to augment their surface wettability. Following the surface preparation, a homogeneous layer of Poly(2,3-dihydrothieno-1,4-dioxin)-poly(styrenesulfonate) (PEDOT:PSS) (Clevios PH, Heraeus) was deposited onto the pristine ITO substrates employing a spin-coating technique at 3000 rpm for 60 s. The spin-coated substrates were subsequently annealed on a hotplate, maintaining at 150 °C for 20 min. The fabrication of the MAPbI_3_ layer was conducted in a nitrogen-filled glovebox. A 1.0 M solution of PbI_2_ in dimethylformamide (DMF) was prepared. This solution was then spin-coated onto the aforeprepared substrates at 6000 rpm for 45 s, followed by a drying phase at 70 °C for 10 min. Subsequently, a solution of methylammonium iodide (MAI) in isopropanol (concentration: 25 mg/mL) was applied over the PbI_2_ layer at 6000 rpm for 45 s. The substrates were then annealed at 100 °C for 40 min.

### Photoconductive atomic force microscopy

Photocurrent Atomic Force Microscopy (Pc-AFM) measurements were conducted utilizing an AIST-NT SmartSPM system under ambient conditions. The apparatus was equipped with gold-coated silicon cantilevers (Budget Sensors ContE-g), characterized by a spring constant of 0.2 N/m, a resonance frequency of 13 kHz, and a probe tip radius of less than 25 nm. The perovskite films were illuminated from the rear through the ITO/glass substrate, employing focused illumination provided by a solar simulator. The procedure for PPIV measurements entailed the positioning of the Pc-AFM probe onto the surface of the sample, applying a consistent force of 5 nN. Subsequently, a loop of *I*–*V* curves was recorded. Following each recording, the probe was retracted, and this sequence was repeated across designated regions of the sample. These regions were scanned at pixel intervals of 20 nm to ensure comprehensive coverage and analysis. The acquisition of *I*–*V* curves was achieved by sweeping the applied voltage from + 1.5 to − 1.5 V, with the sweep duration set at 300 ms and the voltage increment steps maintained at 10 mV. Photovoltaic parameters, which were derived from the analysis of the *I*–*V* curves, were subsequently visualized in pixel arrays using proprietary software.

### Piezoresponse force microscopy

Ionic motion mapping was performed with an AIST-NT SmartSPM in ambient conditions under solar simulator light illumination. Pt-coated cantilevers (Budget Sensors HQ:NSC18/Pt) featuring a spring constant of 2.8 N/m and a resonance frequency of 75 kHz were used. Unless specified otherwise, the conductive probe was subjected to an AC bias of 2 V amplitude and 0.8 kHz frequency. During PFM measurements, the probe maintained contact with the sample surface, exerting a 5 nN load. The sample’s ionic motion response was determined by measuring both lateral (torsional) oscillation amplitudes of the cantilever at the second harmonic frequency (1.6 kHz) in relation to the applied AC voltage.

## Results and discussion

**Fig. 1:**
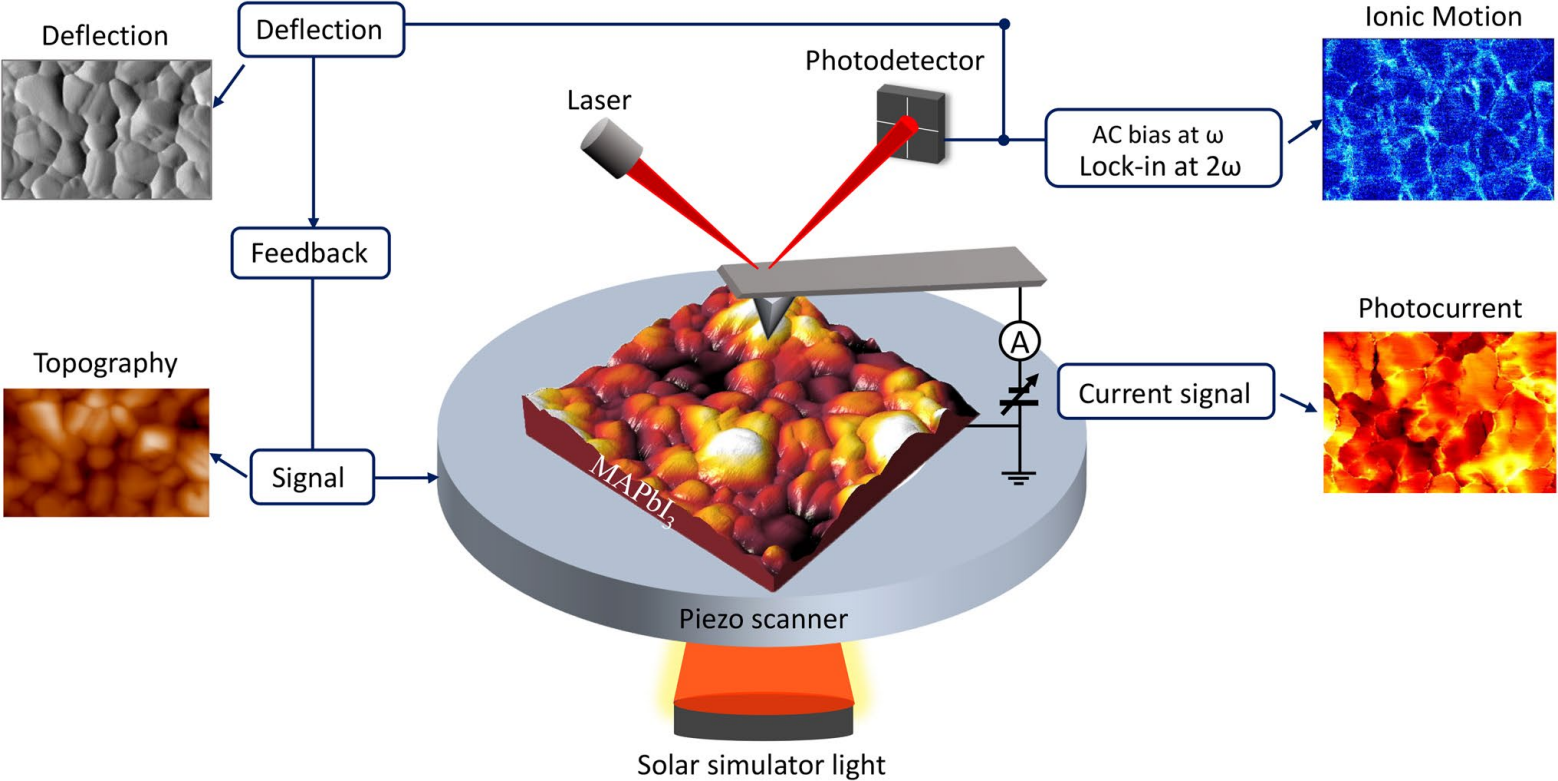
Schematic representation illustrating the multimodal mapping of topography, cantilever deflection, photocurrent, and ionic motion in scanning probe microscopy. Topographical variations result from the expansion or contraction of the piezo scanner throughout the scanning process. The cantilever deflection signal delineates the deviation of the deflection from the predetermined setpoint. Photocurrent images depict the sample’s surface current signal while scanning under illumination. The ionic motion image captures the cantilever’s torsional oscillation signal at the second harmonic frequency in relation to the applied AC bias

Figure 1 presents a schematic illustration of the multimodal mapping of a perovskite thin film using atomic force microscopy (AFM). Illumination of the sample was achieved from below via the transparent electrode. The PEDOT: PSS-coated ITO substrate acts as the bottom electrode for hole collection, while the conductive AFM probe functions as a movable top nanoelectrode for electron collection. A perovskite thin film, fabricated with a two-step inter-diffusion method, is sandwiched in between. Simultaneous acquisition of topography, cantilever deflection, and photocurrent images is achieved as the conductive probe slides across the surface in contact mode, maintaining constant probe-sample interaction. Both topography and cantilever deflection images provide information on surface morphology. Cantilever deflection captures the deviation of the deflection signal from the setpoint, revealing fine surface structures. The photocurrent map documents the current or photocurrent as a function of applied bias during raster scanning across the sample. Ionic motion signals are acquired using second harmonic PFM when the probe, with an AC bias applied, contacts the sample. An oscillating electric field induces ionic motion and subsequent sample surface deformation, detectable by monitoring cantilever motion. Note that the electron transport layer is excluded to directly probe the photovoltaic properties and hysteresis of the bare perovskite thin film via Pc-AFM.

**Fig. 2:**
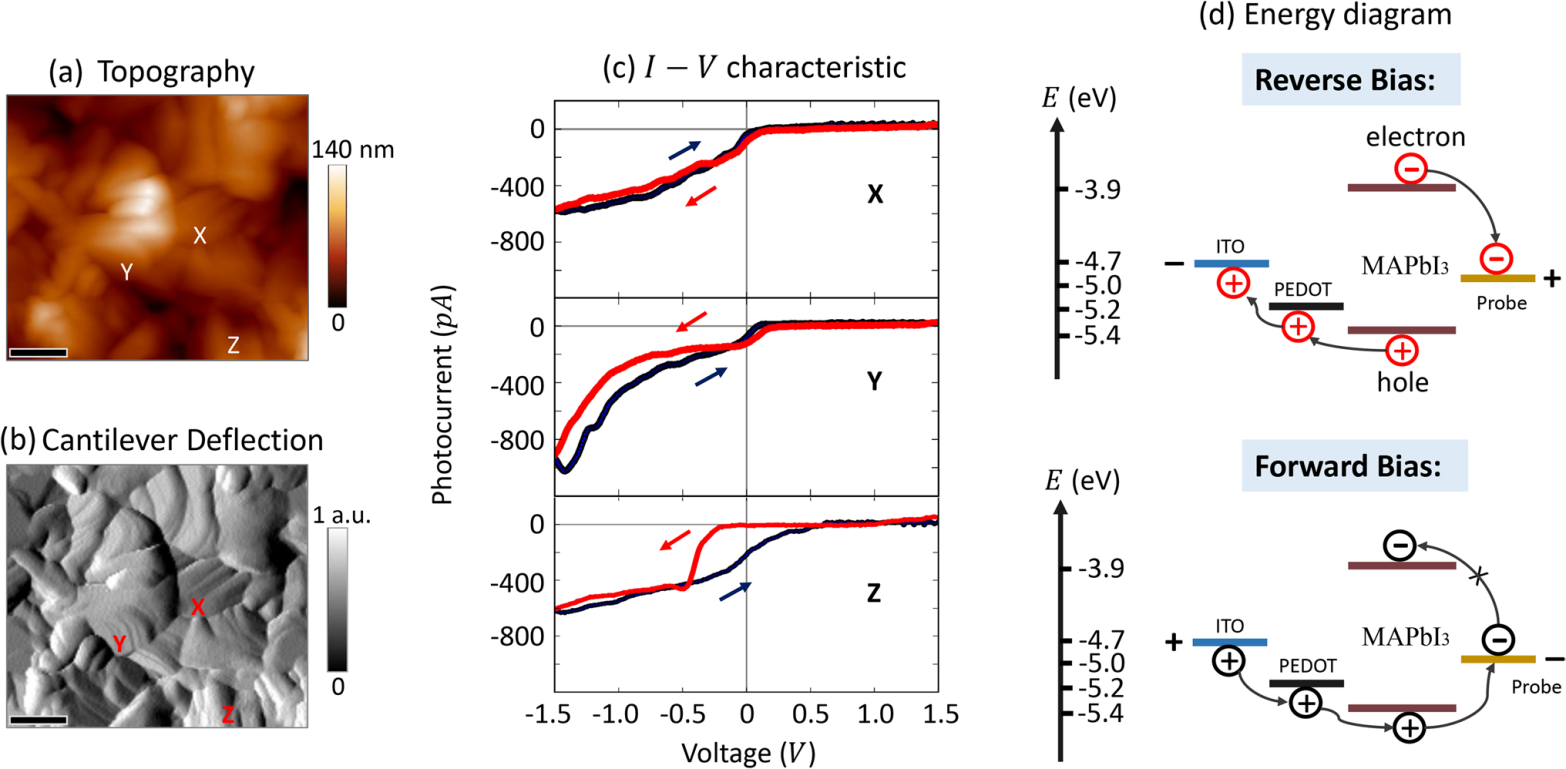
Simultaneously acquired Topographic image of MAPbI_3_ film (**a**) and cantilever deflection signal (**b**). The scale bars correspond to 200 nm. **c** Local *I*–*V* characteristic curves were recorded in a loop, transitioning from a forward bias of + 1.5 V to a reverse bias of − 1.5 V, at three designated locations as indicated in (**a**, **b**). **d** Energy level diagrams illustrate the transport of charge carriers to electrodes under reverse bias (top) and forward bias (bottom). The energy levels are derived from references [44–46]

Figure 2a delineates the surface topography of a methylammonium lead iodide (MAPbI_3_) thin film, as revealed through contact mode AFM. The image elucidates surface features with heights ranging from 0 to 140 nm, and identifies perovskite grains varying in size from 100 to 600 nm. The root-mean-square (RMS) surface roughness is quantified to be 18 ± 3 nm. In Fig. 2a, b deflection image accentuates the variance in cantilever deflection from the established contact mode, thereby providing augmented sensitivity to subtle topographical nuances, surpassing that of the height image.

Figure 2c presents the localized *I*–*V* characteristics under illumination at three designated sites, as referenced in Fig. 2a, b. The forward scan (FS), depicted by a dark blue line, transitions from a negative reverse bias to a positive forward bias, traversing through both short-circuit and open-circuit states. In contrast, the reverse scan (RS), illustrated in red, follows an inverse trajectory. These *I*–*V* curves are derived from cycling the sample bias from + 1.5 V (forward bias) to − 1.5 V (reverse bias), with voltage increments of 10 mV and a sweep cycle duration of 300 ms, corresponding to a sweep rate of 20 V/s. Notably, these measurements were conducted without any preceding bias conditioning or deviation from the open-circuit condition.

Within the first quadrant, the observed *I*–*V* curves exhibit a gradual increment in current rather than the anticipated exponential escalation, indicative of significant series resistance. This phase entails the injection of electrons and holes into the perovskite film from the probe and the bottom electrode, respectively. An appreciable energy barrier for electron injection from the probe, quantified at approximately 1.1 eV as delineated in Fig. 2d, may account for the reduced current observed under forward bias conditions.

In the realm of photoresponse, specifically within the fourth quadrant of Fig. 2c, the local *I*–*V* curves manifest a linear trajectory accompanied by a low fill factor (FF), paralleling findings in organic photovoltaic films assessed through similar Pc-AFM methodologies [41, 47]. It is noteworthy that the *I*–*V* curves at three distinct loci display hysteresis, with the RS exhibiting a more pronounced photoresponse relative to the FS. This phenomenon is reflective of the behavior observed in *J-V* curves of macroscopic solar cells (refer to Additional file 1: Fig. S1).

In the third quadrant, under reverse bias, distinct variations in the *I*–*V* curves are observed at specified sites. Sites X and Y denote grain boundary (GB) regions, with X representing a singular GB location and Y indicating a GB junction point. At site X, current approaches saturation, reaching approximately − 600 pA at − 1.5 V, while at site Y, current significantly escalates with reverse bias, peaking at around − 1000 pA at − 1.5 V. Typically, photocurrent reaches saturation under high reverse bias as photogenerated carriers are efficiently swept towards the electrodes for collection. Nonetheless, the augmented work function of the Au probe may promote hole injection from the probe into the perovskite under reverse bias, as depicted in Fig. 2d. Moreover, the distinctive tip-plane configuration during Pc-AFM might induce a pronounced potential drop at the tip-sample interface [48, 49], thereby facilitating enhanced charge injection. Consequently, the measured current under illumination via Pc-AFM could encapsulate both photocarrier collection and charge injection phenomena, with both contributing to the direction of the observed current [47]. Prior research has associated grain-dependent photoresponse and photoluminescence disparities with variations in trap state densities [32, 37], implying that local charge carrier dynamics could be modulated by nonstoichiometry, which in turn influences variations in surface trap state densities and impacts local energy band alignment [31, 50, 51].

Within the context of nanoscale hysteresis observed in the photoresponse regime, localized *I*–*V* curves obtained at distinct spatial coordinates, denoted as X and Y, manifest normal hysteresis phenomena characterized by curves from the RS exhibiting augmented photocurrent. Conversely, the data procured from location Z indicate the presence of an anomalous, or inverted, hysteresis effect. In an endeavor to systematically map the nanoscale hysteresis phenomenon and elucidate its correlation with the underlying microstructure, local *I*–*V* curve loops were meticulously recorded employing the PPIV technique, subsequently facilitating the generation of detailed hysteresis maps.

**Fig. 3:**
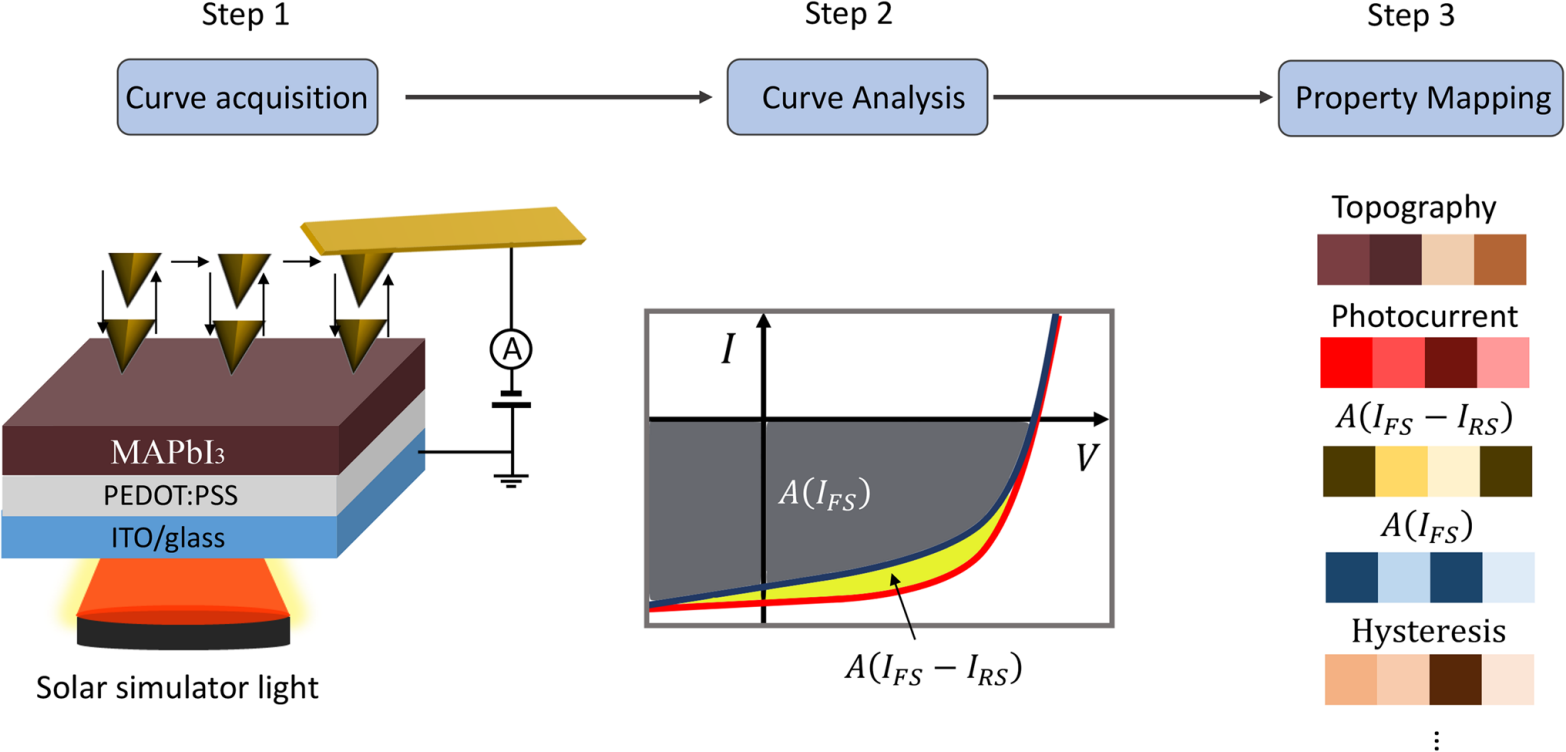
Schematic showing the PPIV technique during Pc-AFM measurements and procedure for constructing hysteresis maps. Step 1 involves sample configuration and AFM setup to acquire arrays of local *I–V* curve loops; step 2 includes curve extraction and analysis using house-built software, including the definition of hysteresis attributes; step 3 entails plotting of hysteresis property maps with color scale

Figure 3 delineates a schematic overview of the PPIV methodology alongside the procedural framework for the construction of hysteresis maps. The initial phase, Step 1, is dedicated to the sample preparation and the precise calibration of the Pc-AFM system, thereby enabling the collection of localized *I*–*V* curve arrays. Proceeding to Step 2, this stage involves the extraction and comprehensive analysis of these curves through the utilization of bespoke software, which notably includes the accurate identification of hysteresis attributes. Culminating in Step 3, this process results in the creation of hysteresis property maps, visually encoded with a color scale to intuitively convey the variance in hysteresis behavior across the sample.

The experimental procedure yielded arrays of *I*–*V* curve loops across 2300 pixel positions (arranged in a 50 × 46 grid) with a spatial resolution of 20 nm between each pixel. Notably, at locations X and Y (also depicted in Fig. 2), the observed photocurrent levels under both scanning directions were found to be comparable, indicating a minimal hysteresis effect. In contrast, at position Z, the FS photocurrent was discernibly higher than that obtained from the RS at a specified reverse bias, thereby unveiling a pronounced hysteresis effect at this particular site.

**Fig. 4:**
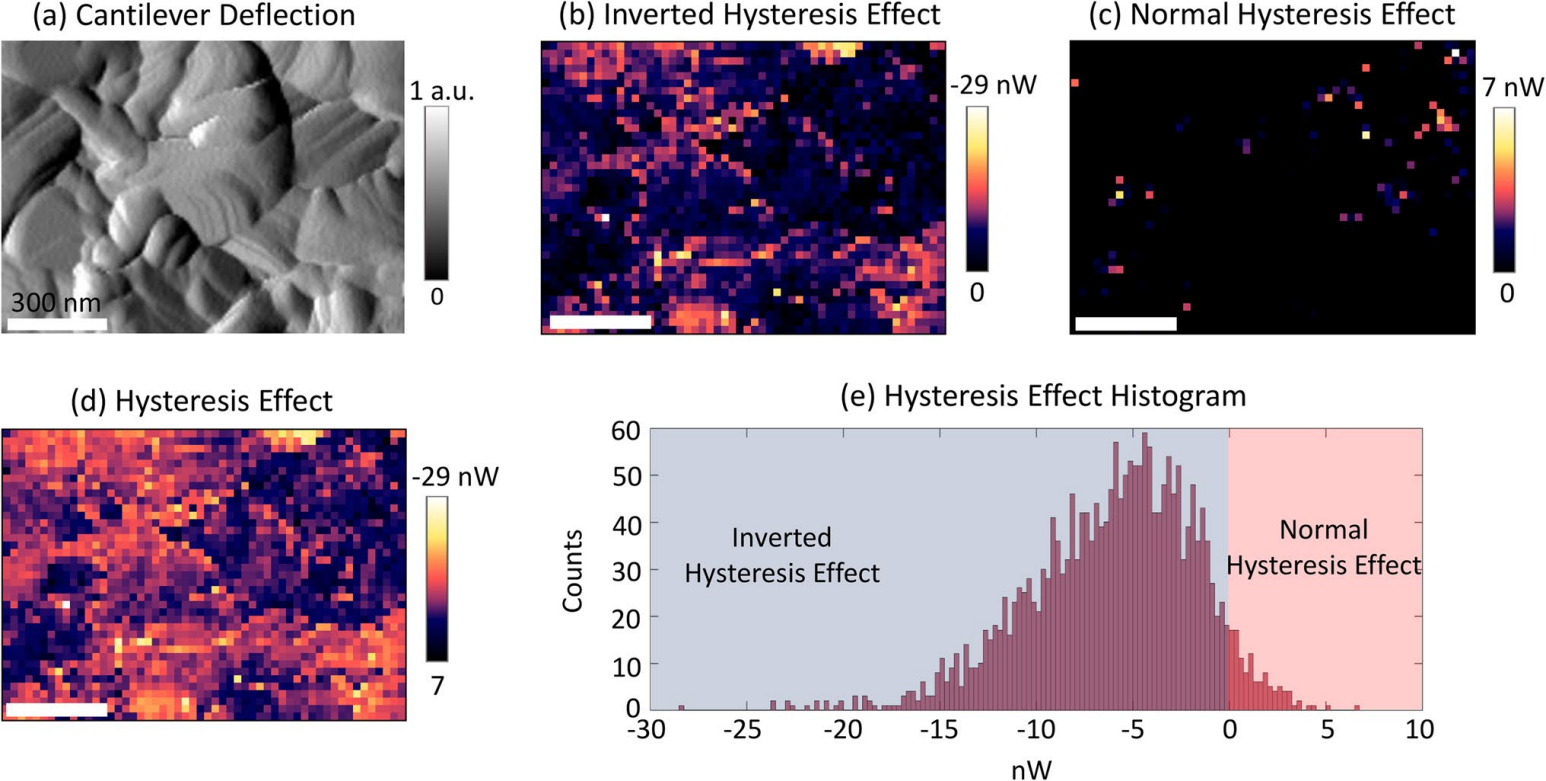
Cantilever deflection map (**a**), constructed inverted Hysteresis Effect map (**b**), normal Hysteresis Effect map (**c**), and Hysteresis Effect image including both inverted and normal hysteresis effect (**d**), HE histograms (**e**) corresponding to **b** shown in grey and **c** in red. The scale bars represent 300 nm

To visualize the spatial variation of hysteresis, Fig. 4 presents a comprehensive visualization of the data obtained through the application of PPIV techniques, specifically focusing on the analysis of hysteresis effects at the nanoscale. Figure 4a illustrates a map of cantilever deflection, providing insights into the topographical variations across the sample surface. Figure 4b depicts a meticulously constructed map of the inverted hysteresis effect, while Fig. 4c showcases a corresponding map for the normal hysteresis effect. Additionally, Fig. 4d amalgamates the observations from Fig. 4b, c, offering a composite hysteresis effect image that encapsulates both inverted and normal hysteresis phenomena. Complementing these visual representations, Fig. 4e features histograms of the hysteresis effect, with the data corresponding to the inverted hysteresis effect in Fig. 4b rendered in grey, and the data pertaining to the normal hysteresis effect in Fig. 4c depicted in red. Each panel is uniformly scaled, with scale bars denoting a measurement of 300 nm, thereby facilitating a comparative analysis of the hysteresis effects observed across different regions of the sample.

The Hysteresis Effect (HE) was delineated as the discrepancy in the integrated area beneath the FS and RS segments of an *I*–*V* curve across a predefined voltage spectrum. Consequently, the mathematical expression for HE is articulated as

1 $${\text{HE}} = A\left( {I_{FS} - I_{RS} } \right)$$

where $I_{{RS\left( {FS} \right)}}$ symbolizes the current corresponding to the RS (FS), and A() denotes the area under the curve, explicitly defined by the integral

2 $$A\left( I \right) = \int {I\left( V \right)dV} .$$

Accordingly, a positive value of HE is indicative of normal hysteresis, whereas a negative HE value signifies the occurrence of inverted hysteresis [5, 52]. As previously elucidated, the manifestation of hysteresis varies significantly across different spatial locations.

To effectively illustrate the spatial heterogeneity of hysteresis, mappings of normal HE and inverted HE were conducted independently. Despite the HE values spanning a range from − 29 to 7 nW, a predominant inclination towards negative (inverted hysteresis) values was observed, with approximately 87% of the total pixel locations exhibiting negative HE values and an average value of − 5.8 ± 0.3 nW. This observation is in alignment with prior findings, indicating that the distribution of HE does not strictly conform to the topographical characteristics of the surface, thereby suggesting a nuanced relationship between hysteresis phenomena and the underlying surface structure. Notably, locations identified as X and Y did not exhibit a pronounced HE.

The magnitude of HE serves as a critical indicator of the degree of local hysteresis and types of hysteresis, which, in turn, significantly influences the overarching macroscopic device hysteresis. Macroscopic devices have been reported to exhibit inverted hysteresis [52, 53], often accompanied by unfavorable band alignment that creates a barrier for charge extraction at the interface and hinders charge transport. Therefore, our findings demonstrate the close relationship between interfacial energetic barriers and the local current–voltage characteristics, which ultimately affect the occurrence of hysteresis. Thus, the prevalent inverted hysteresis emerges as a pivotal determinant of performance in macroscopic solar cell applications, underscoring the importance of understanding and characterizing these local hysteresis effects for the optimization of device functionality.

In the pursuit of a more nuanced quantification of hysteresis relative to the overall signal magnitude within the context of *I*–*V* characteristics, an additional metric, termed the Hysteresis Index (HI), was proposed and methodically defined. The HI is derived by calculating the absolute difference in the integrated areas beneath the FS and RS segments of an *I*–*V* curve across the recorded voltage interval. This calculation diverges from aforementioned approach by aggregating the areas between the two curves rather than allowing them to negate each other, thereby emphasizing the total extent of hysteresis without regard to its directional bias. Subsequently, this absolute disparity is normalized by the larger of the two areas (either FS or RS), to ensure that the HI represents a proportionate measure of hysteresis in relation to the predominant signal contribution. Mathematically, the HI is expressed as

3 $${\text{HI}} = A\left( {|I_{FS} - I_{RS} |} \right)/A\left( {I_{FS or RS} } \right)$$

where A() denotes the area under the curve, $|I_{FS} - I_{RS} |$ signifies the absolute difference in current between the forward and reverse sweeps, and $I_{FS or RS}$ indicates the current corresponding to either the FS or RS, contingent upon which yields the larger area. This formulation of the HI facilitates a comparative analysis of hysteresis effects, providing a standardized index that encapsulates the relative magnitude of hysteresis within the electronic behavior of perovskite solar cells. The HE elucidates the types of hysteresis, e.g., normal hysteresis or inverted hysteresis, and serves as a descriptor of the hysteresis behavior observed in perovskite thin films. The HE identifies the presence and nature of hysteresis in the device’s *I*–*V* characteristics, whereas the HI offers a numerical value that quantifies the magnitude of this hysteresis. By calculating the HI, we can assess the total extent of hysteresis across the voltage spectrum, providing a metric for comparing the hysteresis behavior under different conditions or in different materials. Thus, the HI enables a deeper understanding of the factors influencing hysteresis in perovskite solar cells.

**Fig. 5:**
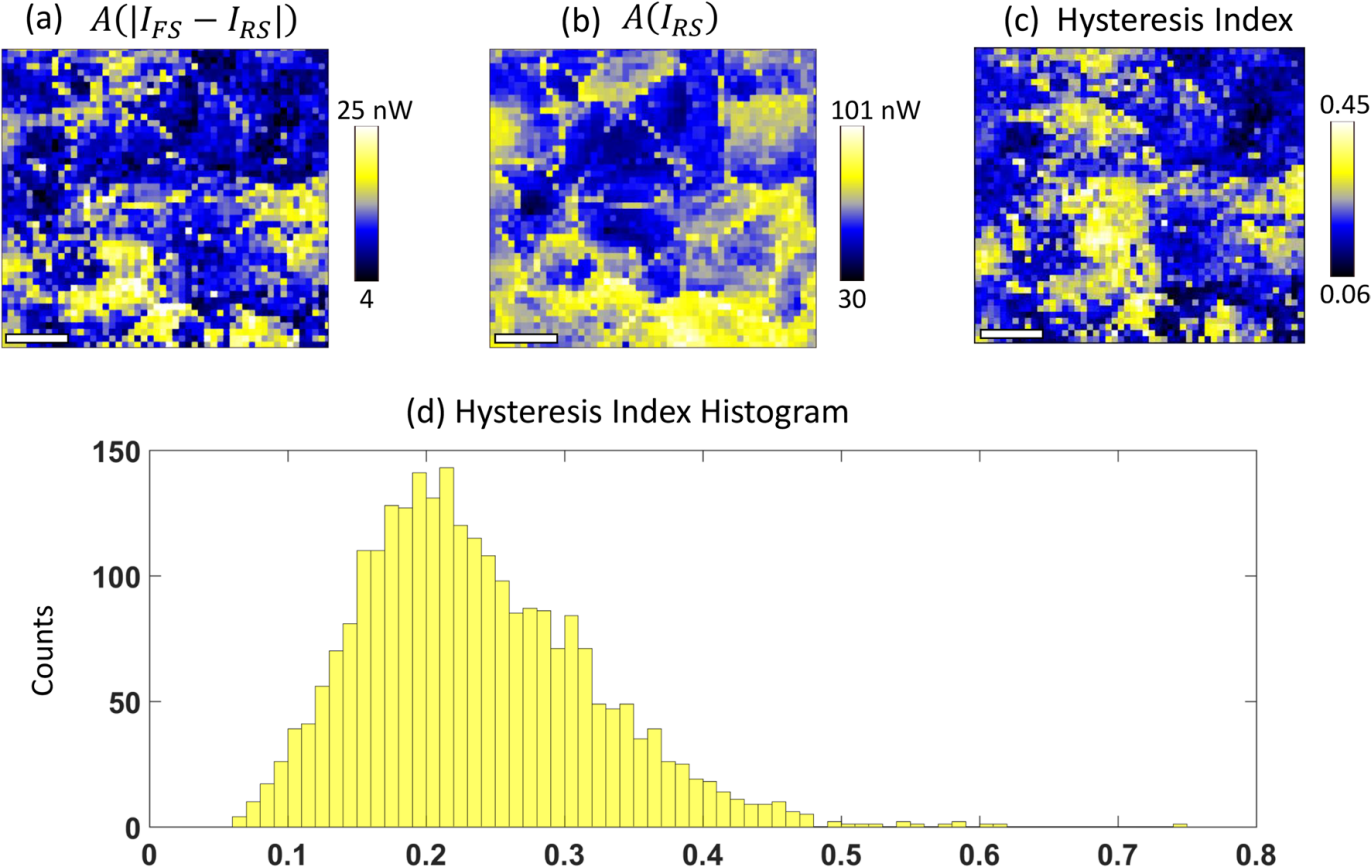
Constructed absolute disparity map between the area under the FS and RS of arrays of *I–V* curves (**a**), current area map under RS (**b**), Hysteresis Index map (**c**) calculated as the disparity map normalized by RS current area map, and HI histogram (**d**). The scale bars represent 200 nm

Figure 5 provides a visualization of the analysis to elucidate the HI characteristics within the sample. Figure 5a illustrates a meticulously constructed map depicting the absolute disparity between the integrated areas under the FS and RS of the *I*–*V* curves, effectively quantifying the extent of hysteresis across the array. Figure 5b presents a current area map derived under the conditions of the RS, offering insights into the distribution of current across the investigated area under specified conditions. Figure 5c, an HI map is showcased, which is calculated by normalizing the disparity map (as shown in Fig. 5a) by the RS current area map (as depicted in Fig. 5b), thereby providing a normalized measure of hysteresis intensity relative to the current distribution. Figure 5d features a histogram of the HI, facilitating a statistical analysis of the hysteresis distribution within the sample. HI values ranges from 0.06 to 0.45 with a mean value of 0.24 ± 0.08. In contrast to the HE image, locations Y and Z in HI exhibit increased hysteresis, with average HI values of 0.39 and 0.31 (calculated over 10 pixels), respectively. On the other hand, locations X show minimal hysteresis, with an HI value of 0.16. Those images aids in the comprehensive understanding of hysteresis phenomena at the nanoscale, highlighting the spatial variability and intensity of hysteresis within the examined arrays.

**Fig. 6:**
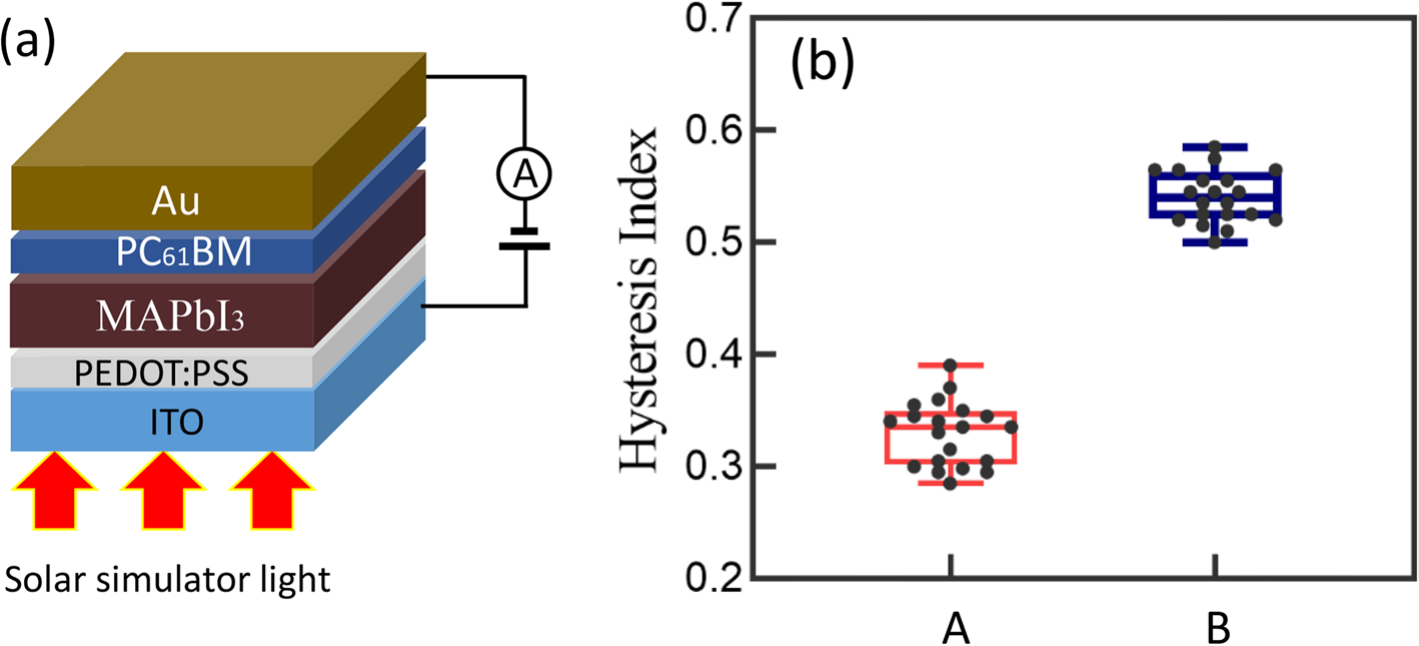
**a** Schematic drawing of perovskite solar cell device configuration for hysteresis characterization, **b** box plot of calculated HI, in which A represents the absolute disparity between the integrated areas under the FS and RS of the *I*–*V* curves, normalized by the current area under the RS and B shows the absolute disparity between the integrated areas under the FS and RS of the *I*–*V* curves, normalized by the current area under the FS

In order to establish a comparative analysis with the macroscopic device hysteresis observed, the HI was investigated on fully assembled perovskite solar cell devices, which incorporate an electron transport layer as a fundamental component of their architecture. This examination is visually represented in Fig. 6a, where the schematic delineation of the device configuration explicitly highlights the inclusion of the electron transport layer. The incorporation of this layer is critical, as it plays a pivotal role in facilitating the efficient movement of electrons within the device and diminishing the energetic barrier for electron collection at the electrode/transport layer interface, thereby influencing its overall performance characteristics, including the manifestation of hysteresis.

In Fig. 6b, two distinct methodologies for the quantification of the HI are delineated, as denoted by labels A and B. These methodologies are predicated upon the analysis of *I*–*V* curves procured from the device, which were obtained under identical conditions of voltage sweep rate and range, mirroring those utilized in nanoscale characterization efforts. Label A presents the calculated HI, where the absolute disparity between the integrated areas beneath the FS and RS of the *I*–*V* curves is normalized by the area under the curve during the RS. It provides a measure of hysteresis relative to the reverse sweep performance. Conversely, label B illustrates the calculation of HI where the absolute disparity between the integrated areas under the FS and RS of the *I*–*V* curves, in this instance normalized by the area under the curve during the FS. This alternative normalization offers a complementary perspective on hysteresis, emphasizing its impact relative to the forward sweep characteristics of the device. Quantitatively, label A exhibits an average HI of 0.34, whereas label B demonstrates an average HI of 0.54. The consistently higher HI values associated with label B, as compared to those of label A, suggest a pronounced increase in the current area under the RS. This observation diverges from the hysteresis characterization at the nanoscale; for macroscopic devices, the *I*–*V* curves obtained from the RS exhibit superior performance, thereby manifesting normal hysteresis behaviors. This finding further corroborates the hypothesis that the previously observed inverted hysteresis phenomena can be attributed to the existence of interfacial energetic barriers.

**Fig. 7:**
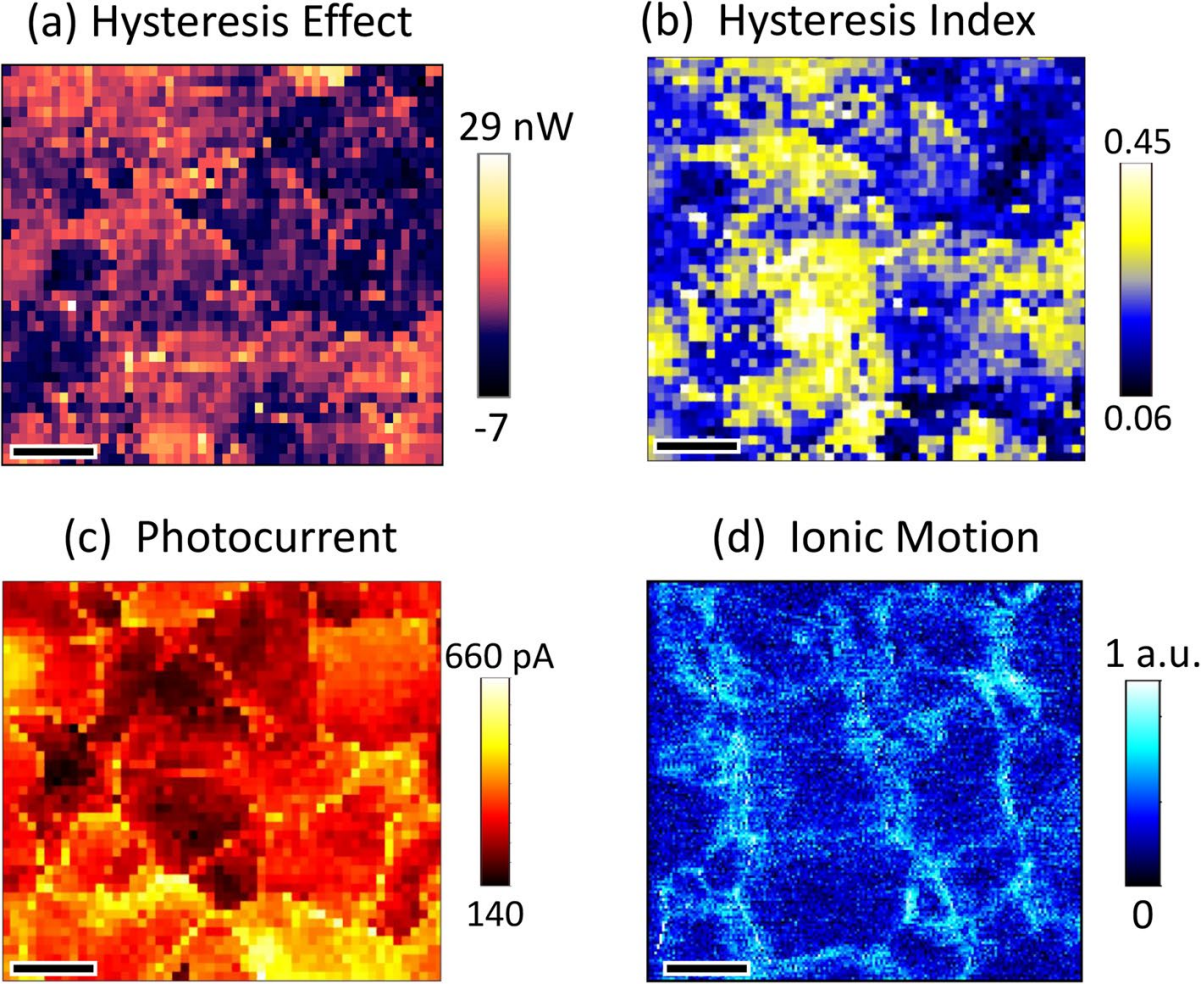
**a** HE map, **b** HI map, **c** photocurrent image under − 0.5 V and **d** ionic motion map for MAPbI_3_ thin film recorded at the same sample location. The scale bars represent 200 nm

In an endeavor to elucidate the potential interrelations among local ion migration, photocurrent, and hysteresis phenomena, a comprehensive analysis was conducted. As presented in Fig. 7, an HE map (Fig. 7a), an HI map (Fig. 7b), a photocurrent map under a reverse bias of − 0.5 V (Fig. 7c), and an ionic motion map (Fig. 7d) are acquired at an identical sample location under illumination. The HE and HI maps, which are reproduced from Figs. 4d and 5c respectively, unveil pronounced heterogeneity in local hysteresis on a grain-to-grain basis.

The photocurrent map under a reverse bias of − 0.5 V, Fig. 7c, demonstrates local variations in photoconduction, indicative of differential charge collection efficiency across the sample. Notably, locations corresponding to GBs exhibit enhanced photoconduction under reverse bias, suggesting that GBs may play a facilitative role in charge transport, thereby implying their benign nature in this context.

The ionic strain map, Fig. 7d, captures the torsional oscillation of the cantilever at the second harmonic frequency of the applied AC bias during PFM. It is postulated that the AC bias induces oscillatory hopping motion of MA^+^ cations along the direction of the electric field, with the inorganic framework undergoing lateral stretching to accommodate this motion [54, 55]. Consequently, the ionic strain map delineates regions where organic cation migration is preferentially initiated under the synergistic influence of both bias and illumination.

As shown in Fig. 7d is that the ionic strain response is predominantly pronounced at and in the vicinity of GBs. This observation aligns with previous studies positing that GBs act as principal conduits for ion migration within polycrystalline perovskite thin films [56, 57]. A direct comparison of the HE, HI, photocurrent, and ionic strain maps (Fig. 7a–d) reveals a lack of direct correlation between the photocurrent and ionic strain signals with either HE or HI. This finding suggests that hysteresis cannot be solely attributed to ion migration or charge collection mechanisms. Instead, the nanoscale HE and HI maps hint at a grain-dependent hysteresis behavior, diverging from a process predominantly associated with phenomena at GBs.

## Conclusions

In conclusion, this investigation has adeptly elucidated the nanoscale photocurrent–voltage hysteresis phenomena within prototype perovskite thin films via a comprehensive examination of localized *I*–*V* curve arrays. Two pivotal hysteresis descriptors have been spatially delineated with nanometric precision. The generated Hysteresis Effect maps predominantly reveal an inverted hysteresis pattern across 87% of the analyzed pixel locations, a phenomenon ascribed to the energetic barrier present at the interface between the probe and the perovskite material. Furthermore, the Hysteresis Index imagery underscores a pronounced heterogeneity and grain-dependent variability, with an average index value of 0.24 identified across the surveyed arrays. Complementary investigations into photocurrent and ionic motion have indicated that the spatial distribution of the two hysteresis descriptors does not correspond directly with either photoconduction or ion migration. These findings imply that the localized hysteresis behaviors captured through Pc-AFM cannot be singularly attributed to either photocharge collection mechanisms or organic cation migration at grain boundaries. Instead, it is posited that the existence of localized surface trap states significantly influences the extraction of photoelectrons and the injection of hole currents at the interfaces between the probe and the sample, thereby engendering a spectrum of hysteresis behaviors.

This research significantly advances the understanding of hysteresis phenomena at the microscale, highlighting the critical role of surface/interface defect trap passivation in mitigating hysteretic effects within perovskite solar cells. Moreover, the refined Pc-AFM methodology introduced herein stands as a versatile tool, offering profound insights into the intricate relationship between photovoltaic performance and nanomorphological characteristics in nascent photovoltaic technologies.

### Supplementary Information

**Additional file 1: Figure S1.** The *J*–*V* curves of the fully assembled perovskite solar cell with architecture ITO/PEDOT: PSS/MAPbI_3_/PC_61_BM/Au. The voltage scan rate is 20 V/s and voltage scan range is from + 1.5 to − 1.5 V. The red curve represents reverse scan and blue curve represents forward scan.

## Data Availability

Data is available from the corresponding author upon reasonable request.
