# Supplementary material for: Unveiling heterogeneity of hysteresis in perovskite thin films
Source: Discov Nano. 2024 Mar 18;19(1):48. doi: 10.1186/s11671-024-03996-9 (PMC10948732; doi:10.1186/s11671-024-03996-9)
Supplement: Supplementary file 1 — Additional file 1: Figure S1. The J–V curves of the fully assembled perovskite solar cell with architecture ITO/PEDOT: PSS/MAPbI3/PC61BM/Au. The voltage scan rate is 20 V/s and voltage scan range is from + 1.5 to − 1.5 V. The red curve represents reverse scan and blue curve represents forward scan. [file 11671_2024_3996_MOESM1_ESM.docx]

**Supporting Information**

**­­­­­­­­­­­** **Unveiling Heterogeneity of Hysteresis in Perovskite Thin Films**

Zhouyiao Zou^1^, Haian Qiu^2^*, Zhibin Shao^1^*

*^1^Industrial Training Center*

Shenzhen Polytechnic University, Shenzhen, Guangdong, 518055, China

*^2^Physics Laboratory, School of Undergraduate Education*

Shenzhen Polytechnic University, Shenzhen, Guangdong, 518055, China

* Correspondence: [qiu20210136@szpu.edu.cn](mailto:qiu20210136@szpu.edu.cn), zhibin_shao@szpu.edu.cn

**1. Macroscopic Device Curves**

**
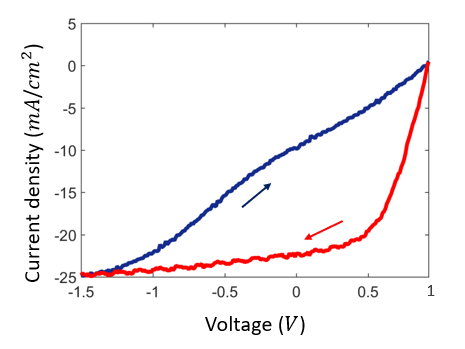
**

Fig. S1. The $J$-$V$ curves of the fully assembled perovskite solar cell with architecture ITO/PEDOT: PSS/MAPbI_3_/PC_61_BM/Au. The voltage scan rate is 20 V/s and voltage scan range is from +1.5 V to -1.5 V. The red curve represents reverse scan and blue curve represents forward scan.
